# Characteristics of and Precipitating Circumstances Surrounding Suicide Among Persons Aged 10–17 Years — Utah, 2011–2015

**DOI:** 10.15585/mmwr.mm6711a4

**Published:** 2018-03-23

**Authors:** Francis B. Annor, Marissa L. Zwald, Amanda Wilkinson, Mike Friedrichs, Anna Fondario, Angela Dunn, Allyn Nakashima, Leah K. Gilbert, Asha Z. Ivey-Stephenson

**Affiliations:** ^1^Epidemic Intelligence Service, CDC; ^2^Division of Violence Prevention, National Center for Injury Prevention and Control, CDC; ^3^Division of Health Nutrition Examination Surveys, National Center for Health Statistics, CDC, Washington, D.C.; ^4^Child Health and Mortality Prevention Surveillance, Center for Global Health, CDC; ^5^Utah Department of Health.

In 2015, suicide was the third leading cause of death among persons aged 10–17 years (*1*), and in Utah, the age-adjusted suicide rate was consistently higher than the national rate during the past decade (*2*). In January 2017, the Utah Department of Health (UDOH) invited CDC to assist with an epidemiologic investigation of suicides among youths aged 10–17 years during 2011–2015 to identify precipitating factors. CDC analyzed data from the Utah Violent Death Reporting System (UTVDRS), National Vital Statistics System, and additional information collected in the field. During 2011–2015 in Utah, 150 youths died by suicide. Approximately three fourths of decedents were male (77.4%) and aged 15–17 years (75.4%). During this period, the unadjusted suicide rate per 100,000 youths in Utah increased 136.2%, from 4.7 per 100,000 population (2011) to 11.1 (2015), whereas among youths nationwide, the rate increased 23.5%, from 3.4 to 4.1. Among suicide decedents with circumstances data available, more than two thirds (68.3%) had multiple precipitating circumstances, including mental health diagnosis (35.2%), depressed mood (31.0%), recent crisis (55.3%), and history of suicidal ideation or attempt (29.6%). CDC’s technical package of policies, programs, and practices to prevent suicide supported by the best available evidence can be used as a suicide prevention resource (*3*).

UTVDRS is part of CDC’s National Violent Death Reporting System,* which collects information on violent deaths, including suicides, from multiple sources, including death certificates, coroner and medical examiner reports, and law enforcement reports, to monitor trends, understand violent death characteristics and circumstances, and inform prevention efforts (*4*). Data from the National Vital Statistics System, accessed through CDC WONDER, provided national data for the comparison of suicide rates between Utah and U.S. youths aged 10–17 years during 2011–2015 (*2*). The crude suicide rate per 100,000 was estimated and descriptive analyses were performed to examine the demographic characteristics, precipitating circumstances, and toxicology results of decedents. Joinpoint regression was performed to test trends over time and to estimate annual percentage change. The additional data collected were obtained from medical examiner, law enforcement, autopsy, and toxicology reports, as well as obituary and online news articles. Previous research and initial reading of some of the narratives on youth suicide informed the collection of the additional data, which were considered potential precipitating circumstances for youth suicide, but which are not routinely collected by the UTVDRS, such as cutting and other self-harm behaviors. For this investigation, suicide was defined using the *International Classification of Diseases, Tenth Revision* (ICD-10) underlying cause of death codes X60–X84.

During 2011–2015, 150 youths aged 10–17 years died by suicide in Utah. More than three fourths of these decedents were male (77.4%), non-Hispanic white (81.3%), and aged 15–17 years (75.4%; average age = 15.3 years [standard deviation = 1.6]) (Table 1). The two most common methods of suicide were suffocation and firearm, which accounted for 46.0% and 45.3% of deaths, respectively. Among 148 suicide victims with information on location, 124 (83.8%) of the fatal injuries occurred at home.

**TABLE 1:** Suicides* among persons aged 10–17 years (N = 150), by selected characteristics — Utah, 2011–2015

Characteristic	No. (%)
**Sex**	
Male	116 (77.4)
Female	34 (22.6)
**Race/Ethnicity**	
White, non-Hispanic	122 (81.3)
Nonwhite^†^	28 (18.7)
**Age group (yrs)**	
10–14	37 (24.6)
15–17	113 (75.4)
Mean (SD, range)	15.3 (1.6, 10–17)
**Mechanism**	
Suffocation^§^	69 (46.0)
Firearm	68 (45.3)
Other^¶^	13 (8.7)
Location**	
Home^††^	124 (83.8)
Other^§§^	24 (16.2)

**Abbreviation:** SD = standard deviation.

* *International Classification of Diseases, Tenth Revision* underlying cause of death codes X60–X84.

^†^ Included American Indian, Asian, Black, Hispanic, and Pacific Islander.

^§^ Includes hanging, strangulation, and deaths involving deprivation of oxygen attributable to inhalation of asphyxiant gases (e.g., helium, nitrogen, propane, argon, and butane).

^¶^ Includes poisoning, fall, drowning, and other transportation.

** Two decedents had missing information on location; therefore, N = 148.

^††^ House, apartment, rooming house, including driveway, porch, yard, and garage.

^§§^ Includes farm, natural area, motor vehicle, railroad tracks, office building, park/playground, and street/road.

The unadjusted suicide rate among Utah youths aged 10–17 years increased an average of 22.8% per year during 2011–2015 (p<0.001), with a total increase of 136.2%, from 4.7 per 100,000 population in 2011 to 11.1 in 2015. Nationwide in the United States, the unadjusted suicide rate increased 6.0% per year during this period (p<0.001) (total increase of 23.5% [range for the census regions = -7.1% to 36.8%], from 3.4 per 100,000 in 2011 to 4.2 in 2015). The annual unadjusted suicide rate among Utah youths was higher than the U.S. rate for all years studied (Figure).

**FIGURE:**
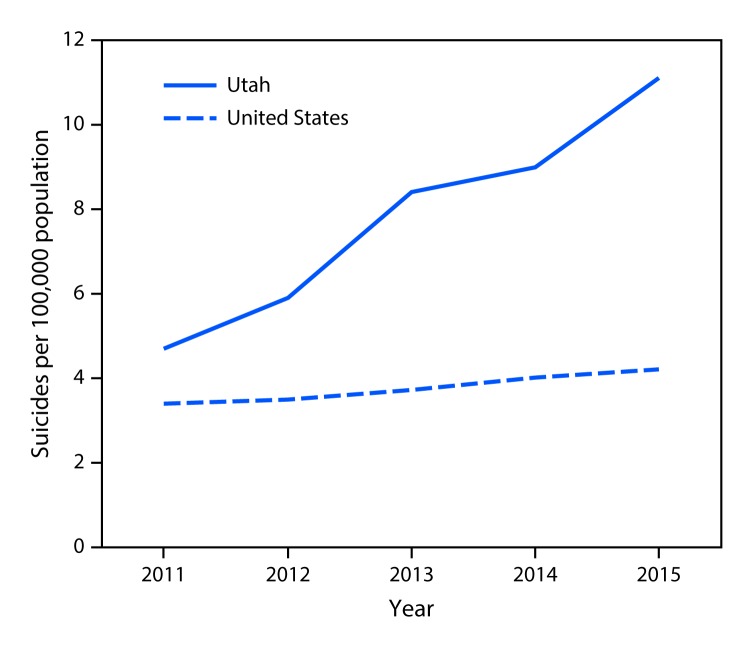
Unadjusted suicide rates among youths aged 10–17 years — Utah* and United States,^^†^^ 2011–2015 **Source:** CDC Vital Statistics data accessed through CDC WONDER. * Annual percentage change (APC) for Utah = 22.8% (p<0.001). ^^†^^ APC for United States = 6.0% (p<0.001).

Among the 142 decedents for whom circumstance information was available, two or more precipitating circumstances were identified before death in 97 (68.3%). Fifty (35.2%) decedents with data had a diagnosed mental health problem, and 44 (31.0%), including 34 (23.9%) who had no mental health diagnosis, were described as being in a depressed mood at or near the time of death. (Table 2). Among the 50 decedents with a mental health diagnosis, 42 (84.0%) were in treatment at the time of death. A history of suicidal ideation, a previous suicide attempt, or both was reported for 42 (29.6%) decedents for whom circumstance information was available. A recent crisis, defined as an event occurring within 2 weeks of death that was indicated to have contributed to the death, was reportedly experienced by 83 (55.3%) decedents; these were most commonly family relationship problems (31, 21.8%) and intimate partner problems (15, 10.6%). Other crises included school problems and suicide of a friend or a family member. Among 131 (92.2%) decedents tested, 26 (19.8%) had one or more of the following drugs detected in their system at the time of death: alcohol, cocaine, amphetamines, marijuana, and opiates. Family conflicts that were the result of or that resulted in technology use restriction (i.e., limitations in the use of technological devices that resulted in family conflict or other family conflicts that resulted in restriction to the use of technological devices such as mobile phones, tablets, gaming systems, or laptops within 7 days before dying by suicide) were reported for 18 (12.7%) decedents. Thirty-four (23.9%) decedents disclosed their intent to die by suicide, 67 (47.2%) left a suicide note, and 30 (21.4%) had a history of cutting or had recently cut themselves.

**TABLE 2:** Precipitating circumstances for suicide* among youths aged 10–17 years (N = 142^ɫ^) — Utah, 2011–2015

Characteristic	No. (%)
**Mental health diagnosis^§^**	50 (35.2)
Mental health treatment among those with a diagnosis^¶^	42 (84.0)
**Current depressed mood**	44 (31.0)
**History of suicidal thoughts/plans or suicide attempt**	42 (29.6)
Suicidal thoughts	26 (18.3)
Suicide attempts	23 (16.2)
**Family relationship problems**	45 (31.7)
**Dating partner problems**	22 (15.7)
Recent crisis**	83 (55.3)
Family relationship problems^††^	31 (21.8)
Intimate partner problems^††^	15 (10.6)
School problem, suicide of friend/family, criminal legal problems^††^	19 (13.4)
Crisis not associated with a circumstance^††^	32 (22.5)
**Disclosed intent** ^§§^	34 (23.9)
To friend^¶¶^	14 (63.6)
To parent/guardian	11 (50.0)
**Left a suicide note**	67 (47.2)
Positive toxicology results***	26 (19.8)
**Family conflicts related to technology use restriction** ^†††^	18 (12.6)
**Cutting or history of cutting** ^§§§^	30 (21.4)
**More than two precipitating circumstances** ^¶¶¶^	97 (68.3)

* *International Classification of Diseases, Tenth Revision* underlying cause of death codes X60–X84.

^ɫ^ Decedents with known circumstances data and excludes missing. Unless otherwise noted, the denominator used to estimate the percentage = 142.

^§^ Disorders included diagnoses such as major depression, schizophrenia, and generalized anxiety disorder, as well as neurodevelopmental disorders (such as intellectual disability, autism, attention-deficit /hyperactivity disorder), eating disorders, personality disorders, and organic mental disorders.

^¶^ Denominator included only persons with diagnosed mental health problems.

** Refers to a current/acute event (within 2 weeks of death) that is reported in one of the source documents to have contributed to the death. The denominator = 150.

^††^ Crises are not mutually exclusive. A decedent might have experienced multiple crises, therefore percentage do not sum to 100%.

^§§^ For 22 of 34 decedents who disclosed their intent, information about person(s) to whom intent was disclosed was available.

^¶¶^ Included a friend, classmate, boy/girlfriend, ex-boy/girlfriend. Percentage do not sum to 100% because decedent might have disclosed intent to multiple persons.

*** Among 131 decedents tested for alcohol, cocaine, amphetamine, marijuana, and opiates.

^†††^ Limitations in the use of technological devices that resulted in family conflict or other family conflicts that resulted in restriction to the use of technological devices such as mobile phones, tablets, gaming systems, or laptops within seven days before dying by suicide.

^§§§^ Denominator = 140.

^¶¶¶^ Estimated using the following circumstance information: Mental health diagnosis, current depressed mood, history of suicidal thoughts or plans, history of suicide attempts, family relationship problems, dating partner problems, recent crisis, and disclosure of intent to die by suicide.

## Discussion

Reports from national data have highlighted increasing suicide rates among adolescents in recent years (*5*). This investigation indicated that the unadjusted suicide rate in Utah among persons aged 10–17 years more than doubled during 2011–2015, while the national rate increased 23.5%. The average annual increase of 22.8% observed in Utah youths was almost four times higher than the 6.0% increase observed in this age group nationwide. Whereas this investigation could not identify specific factors driving the increase in suicide among Utah youths, across multiple data sources, mental health, relationship problems, family conflicts, and experience of other forms of violence were common among Utah youths who died by suicide (https://health.utah.gov/wp-content/uploads/Final-Report-UtahEpiAid.pdf).

The prevalence of precipitating circumstances identified among suicide decedents aged 10–17 years and the proportion experiencing multiple precipitating circumstances are consistent with findings from previous investigations (*6*,*7*). Mental health problems, including depressed mood, were common among suicide decedents. Therefore, improving access to evidence-based mental health care for youths who do not have access might benefit suicide prevention efforts. Also, given that 84.0% of decedents with a mental health diagnosis were in treatment at the time of death, suicide prevention stakeholders and mental health professionals are encouraged to examine existing mental health treatment approaches and their timeliness to ensure they are consistent with the current evidence-based treatment approaches (*8*).

The data on recent crises and circumstances reported for suicide decedents suggest opportunities for prevention, in addition to strategies to promote mental health. For example, that approximately one in five decedents had experienced recent family relationship problems and one in 10 had experienced recent intimate partner problems suggest a lack of connectedness, a sense of belonging, trust, caring, and respect, which might erode safeguards that have been shown to buffer against suicidal behaviors (*9*). This loss of connectedness has been associated with social isolation and a sense of burdensomeness, both of which have been associated with suicidal behaviors in youths (*9*).

Approaches that promote connectedness and teach coping and problem-solving skills, such as peer norms programs, community engagement activities, social-emotional learning programs, and parenting skill and family relationship programs as part of a comprehensive approach, might help prevent suicide among youths in Utah (*3*). Approximately 12.6% of decedents experienced family conflicts as a result of or that resulted in technology use restriction before death. Additional research is needed to understand the implications of this finding, including the extent to which it represents interruption to social support networks, distress over losing access to the device, confounding with the reason for punishment (e.g., poor grades), or other factors.

The findings in this report are subject to at least four limitations. First, because of the small sample size, group differences in trends (by sex and race/ethnicity) could not be examined. Second, information about mental health diagnosis and other circumstances were obtained from medical examiner reports and decedent family but not from medical records, which might have implications for over- or underestimating the true prevalence. Third, information on protective factors are not included in this report because of the nature of the source documents used and their focus on risk factors associated with death. Finally, death certificates might undercount suicide (*10*), and in Utah, the rate of death with undetermined intent is higher than the U.S. average (*2*). It is likely some of the undetermined intent deaths might be suicide; therefore, suicide rate in Utah might have been underestimated in this report.

During 2011–2015, approximately two thirds of youths aged 10–17 who died by suicide in Utah experienced multiple and diverse precipitating circumstances before death. A multicomponent, comprehensive, and coordinated suicide prevention approach that targets multiple precipitating circumstances is important for reducing and preventing suicide in this population. CDC’s technical package of policies, programs, and practices to prevent suicide supported by the best available evidence can be used as a suicide prevention resource (*3*). Strategies to strengthen access and delivery of suicide prevention care, promote connectedness, create protective environments, and teach coping and problem-solving skills as part of a comprehensive suicide prevention effort might benefit Utah youths (*3*).

**Summary**

**What is already known about this topic?**

Suicide is a major public health problem. It is the third leading cause of death among U.S. persons aged 10–17 years. In Utah, the rate of suicide among persons aged 10–17 years has increased since 2011 and is substantially higher than the national average.

**What is added by this report?**

Approximately two thirds of suicide decedents in Utah aged 10–17 years had multiple precipitating circumstances such as mental health problems, depressed mood, family relationship problems, dating partner problems, history of suicidal ideation or attempt, and experience of recent crisis that preceded their death. Approximately one in 10 decedents had experienced a family conflict that resulted in or that was a result of technology restriction before death.

**What are the implications for public health practice?**

Although the reasons for the high rate of youth suicide in Utah are not known, a multicomponent, comprehensive, and coordinated suicide prevention approach that addresses mental health issues, enhances connectedness, and targets multiple precipitating factors could benefit youths at risk for suicide in Utah.

## Footnotes

* https://www.cdc.gov/violenceprevention/nvdrs/index.html.
